# Analysis of education level in access and use of health care services, ISA-Capital, São Paulo, Brazil, 2003 and 2015

**DOI:** 10.1590/0102-311XEN249122

**Published:** 2023-10-09

**Authors:** Edige Felipe de Sousa Santos, Marília Cristina Prado Louvison, Elaine Cristina Tôrres Oliveira, Camila Nascimento Monteiro, Marilisa Berti de Azevedo Barros, Moisés Goldbaum, Chester Luiz Galvão Cesar

**Affiliations:** 1 Faculdade de Saúde Pública, Universidade de São Paulo, São Paulo, Brasil.; 2 Universidade Estadual de Ciências da Saúde de Alagoas, Maceió, Brasil.; 3 Hospital Sírio-Libanês, São Paulo, Brasil.; 4 Faculdade de Ciências Médicas, Universidade Estadual de Campinas, Campinas, Brasil.; 5 Faculdade de Medicina, Universidade de São Paulo, São Paulo, Brasil.

**Keywords:** Socioeconomic Factors, Health Services Accessibility, Health Surveys, Fatores Socioeconômicos, Acesso aos Serviços de Saúde, Inquéritos Epidemiológicos, Factores Socioeconómicos, Accesibilidad a los Servicios de Salud, Encuestas Epidemiológicas

## Abstract

The great socioeconomic inequality that prevails in Brazil and the existence of a national health system with universal coverage places the need to monitor the evolution and social inequities regarding access to these services. This study aims to analyze the changes in the prevalence of health care use and the extent of social inequality in the demand, use and, access, resolution of health problems, satisfaction, and health care use of Brazilian Unified National Health System (SUS) according to education levels in the population living in the urban area of the Municipality of São Paulo, in 2003 and 2015. We analyzed data from two population-based household health surveys (*Health Survey in São Paulo City* - ISA-Capital) from 2003 and 2015. Dependent variables related to health care use in the two weeks preceding the survey and due to diseases included demand, access, satisfaction, problem resolution, and the public or private nature of the service. Prevalence was estimated using level of education and prevalence ratios (PR) by the Poisson regression. In the period, the demand for health care, access, resolution, and use of public health care increased from 2003 to 2015. Inequities in public health care use changed from 2003 to 2015 according to level of education. We found no social inequities in health care use in the municipality of São Paulo regarding demand, access, satisfaction, and resolution according to levels of education. Results show progress in the use and resolution of health care services, as well as the strong concentration of the use of SUS by the population with lower education. Results indicate the progress that SUS has made, but also show persistent challenges in the use and access to services.

## Abstract (pt)

A grande iniquidade socioeconômica que prevalece no Brasil e a existência de um sistema nacional de saúde com cobertura universal torna necessário o acompanhamento da evolução e das iniquidades sociais no acesso aos serviços. Analisar as mudanças na prevalência do uso de serviços de saúde e o grau de iniquidade social considerando a demanda, o uso e acesso, resolução de problemas de saúde, satisfação e utilização dos serviços de saúde do Sistema Único de Saúde (SUS), segundo o nível de escolaridade, na população residente na zona urbana do Município de São Paulo, em 2003 e 2015. Foram analisados dados de dois inquéritos domiciliares de saúde de base populacional (*Inquérito de Saúde do Município de São Paulo* - ISA-Capital) de 2003 e 2015. As variáveis dependentes relacionadas à utilização de serviços de saúde nas duas semanas anteriores à pesquisa e devido à presença de alguma doença incluem: demanda, acesso, satisfação, resolução do problema e a natureza pública ou privada do serviço. A prevalência foi estimada por meio da escolaridade e das razões de prevalência (RP) por regressão de Poisson. Entre 2003 e 2015, a demanda por cuidados de saúde, acesso, resolutividade e utilização de serviços públicos de saúde aumentou. As iniquidades no uso da saúde pública mudaram de 2003 para 2015 quando se trata do nível de escolaridade. Não foram encontradas iniquidades sociais na utilização dos serviços de saúde no Município de São Paulo em termos de demanda, acesso, satisfação e resolutividade, segundo o nível de escolaridade. Os resultados mostram avanços na utilização e resolutividade dos serviços de saúde, bem como uma forte concentração do uso do SUS pela população com menor nível de escolaridade. Os resultados indicam os avanços do SUS, mas também mostram que ainda há desafios no uso e acesso aos serviços.

## Abstract (es)

La gran desigualdad socioeconómica que prevalece en Brasil y la existencia de un sistema nacional de salud con cobertura universal hace necesario el seguimiento de la evolución y de las desigualdades sociales en el acceso a los servicios. Analizar los cambios en la prevalencia del uso de servicios de salud y el grado de desigualdad social considerando la demanda, el uso y acceso, resolución de problemas de salud, satisfacción y utilización de los servicios de salud del Sistema Único de Salud brasileño (SUS), según el nivel de educación, en la población residente en la zona urbana del Municipio de São Paulo, en 2003 y 2015. Se analizaron los datos de dos encuestas de salud domiciliaria de base poblacional (*Encuesta de Salud en el Municipio de São Paulo* - ISA-Capital) de 2003 y 2015. Las variables dependientes relacionadas con el uso de los servicios de salud en las dos semanas anteriores a la investigación y debido a la presencia de alguna enfermedad incluyen: la demanda, el acceso, la satisfacción, la resolución del problema y la naturaleza pública o privada del servicio. La prevalencia se estimó mediante la educación y las razones de prevalencia (RP) mediante regresión de Poisson. Entre 2003 y 2015, aumentó la demanda de atención médica, el acceso, la resolución y el uso de los servicios de salud pública. Las desigualdades en el uso de la salud pública cambiaron de 2003 a 2015 en lo que respecta al nivel de educación. No fueron encontradas desigualdades sociales en la utilización de los servicios de salud en el municipio de São Paulo en términos de demanda, acceso, satisfacción y resolutividad, según el nivel de educación. Los resultados muestran avances en la utilización y la resolutividad de los servicios de salud, así como una fuerte concentración del uso del SUS por parte de la población con menor nivel de educación. Los resultados indican los avances del SUS, pero también muestran que todavía hay desafíos en el uso y acceso a los servicios.

## Introduction

In Brazil, the demand for health care is increasing due to its higher life expectancy, changes in its age structure pyramid, and the triple burden of diseases (chronic diseases, infectious diseases, and external causes) ^1^ ^,^ ^2^. This growing demand for health care pressures its health system to reorganize supply to meet the needs of its population and address one of the greatest challenges of the Brazilian Unified National Health System (SUS): access to services in the face of social inequities ^2^.

Brazil is the only country with a population of more than 200 million inhabitants that has a universal system covering health care, health surveillance, drug supply, research and development, and free vaccinations, among other services, from primary care to highly complex treatments ^3^. In its years of implementation, SUS has struggled with challenges related to its size but it has shown significant results regarding service and professional delivery as well as changing the pattern of health care use ^4^.

Use of health care services and access to them rely on the interplay of factors (geographic, economic, social, racial, and gender) that determine the use of services by different populations ^5^. These factors (which related to both the perceived need and availability of services) play an important role in demand and are used as elements of analysis to generate evidence on social inequities ^6^.

Health care use is an important element of analysis in health surveys because it provides information about demand and factors associated with service use ^7^. Population surveys that have reviewed health care use and its typology in the general population ^8^ ^,^ ^9^ ^,^ ^10^ or (even in specific populations such as immigrants, women, and seniors ^11^ ^,^ ^12^ ^,^ ^13^) have found that socioeconomic, demographic, and demand factors act as predictors of service use. In Brazil, a survey has attempted to examine both changes over time ^14^ ^,^ ^15^ ^,^ ^16^ ^,^ ^17^ and social inequities in the population’s use and access to health care. A study that sought to compare the demand and health care use in Brazil from 2013 to 2019, based on data from the *Brazilian National Health Survey* (PNS), found an increase of about 22% in health care use in the two weeks preceding the survey over the studied period (from 15.3% in 2013 to 18.6% in 2019). Another important finding is that the analysis of all attempts of receiving health care showed a decrease of 11% among those who sought care (from 97% in 2013 to 86.1% in 2019) ^17^.

Measuring access to health care systems and services and their use is an important object of study as it indirectly offers the possibility of assessing attributes associated with equity ^18^. Thus, we emphasize the need to know the current pattern of social inequities in health and to estimate their magnitude to contribute to the literature on the subject and to the planning and management of community health interventions that prioritize the most vulnerable groups to reduce the found health inequities and discuss the success of the implemented interventions. This study aimed to analyze changes in the prevalence of access and use of health care services for the diseases reported in the two weeks preceding the aforementioned survey, as well as the extent of social inequality in demand, use, and access, resolution of health problems, satisfaction, and use of health care services from SUS, according to levels of education in the population living in the urban area of the Municipality of São Paulo in 2003 and 2015.

## Materials and methods

### Design

This is a population-based health survey conducted in 2003 and 2015 in the Municipality of São Paulo (ISA-Capital). It was developed by a team of researchers from the School of Public Health and the Medical School of the University of São Paulo (FSP and FM/USP), the School of Medical Sciences from the University of Campinas (FCM/UNICAMP), and the Health Institute of the Department of Health of São Paulo (IS/SES-SP). The main objective of the ISA-Capital survey was to diagnose the population’s living and health conditions and health care use. Methodological details of the ISA-Capital outputs can be found on the ISA-Capital website (https://www.prefeitura.sp.gov.br/cidade/secretarias/saude/epidemiologia_e_informacao/isacapitalsp/) and in the previous publications ^19^ ^,^ ^20^ ^,^ ^21^ ^,^ ^22^.

### Populations and sampling process

Sampling was conducted in two stages: census tracts and households. All individuals aged ≥ 20 years were included. The sample in 2003 included 1,667 individuals and in 2015, 3,184 individuals. All respondents were interviewed, informed, and agreed to participate in the study. This study was approved by the Ethics Committee of the FSP/USP (protocol: 719.661/2014).

### Variables

The following socioeconomic and demographic variables were selected: gender (male and female), age (20-59 years, 60 years or more), race/color (white, black/mixed-race, and yellow), level of education in full years (0-7, 8 and more), presence of noncommunicable chronic disease (NCD) (yes, no), and self-perceived health (excellent/very good, good, bad/very bad). Socioeconomic and demographic variables were used to characterize the population of this study.

Health problems in the two weeks preceding the survey, demand for health care, access and use of health care services, satisfaction with the care received at the health service, resolution of the health need among those who resorted to health care services, and use of public health care were adopted as the variables of interest in this study. As the 2003 and 2015 ISA-Capital surveys indirectly assessed quality of care, two closely related measures were used to assess quality of care: satisfaction regarding the received treatment and resolution of health problems ^23^.

To assess access and use of health care services, the following questions from the ISA-Capital Questionnaire were considered: “Health problem”, to assess health problems in the two weeks prior to the survey (C101a. Did you have any health problems in the last 2 weeks?); “Demand for health care”, to assess the demand for health services to solve this health problem (C101i. Did you seek help or talk to someone to solve this health problem?); “Access and use of health care”, to assess access to and use of health care services at the sought service (G111. Did you receive care at the service you sought?); “Satisfaction”, to assess satisfaction with the care received at the health service (G134. What did you think of the care you received?); “Resolution”, to assess the resolution of the health need among those who resorted to health care (G137. Has your need been resolved?); and “Use of the public health service”, to assess the use of the public health service in the city of São Paulo (G110. Is this health service public or private/private?).

Health problems in the two weeks prior to the survey considered all illnesses or health problems that the person had in the two weeks, whether acute or chronic diseases. Thus, such diseases or health problems may have emerged in those 14 days prior to the interview or a long time before it. Although people are more likely to mention problems that began in the last two weeks, they can refer to any chronic problems they have to answer this question.

### Statistical analysis

First, the individuals in the sample were characterized according to the considered variables, using absolute frequencies and relative percentages in the weighted sample. Prevalence ratios (PR) were estimated by Poisson regression. The prevalence of health care use - outcome variable - was analyzed by demand, access, resolution, satisfaction, use of SUS services, whereas the independent variable consisted of education level in whole years (reference category: eight years or more of schooling) to determine the extent of inequality in health care use for acute conditions. Moreover, the variables gender and age were used as adjustments in the regression models to control for potential confounders. A descriptive level of 0.05 was considered for the Wald test.

All analyses were performed using the *survey* module of Stata 14.0 (https://www.stata.com), considering the complex effects of our study design and the effect of stratification and embedding the different observation weights.

## Results

Table 1 shows the distribution of the population by the following variables: demographic characteristics, socioeconomic characteristics, and health conditions in 2003 and 2015. Regarding demographic characteristics, we found that the proportion of individuals aged 60 years or more tended to grow between the studied years, from 15.99% in 2003 to 18.51% in 2015. Regarding socioeconomic characteristics, we found that between 2003 and 2015, the proportion of individuals who identified themselves as white decreased (67.51% and 52.41%, respectively). We also observed a decrease in the number of individuals with up to three years of schooling (14.16% in 2003 and 6.84% in 2015) and an increase in those who reported 12 and more years of schooling (23.93% in 2003 and 28.75% in 2015).

**Table 1:** Characterization of the study population by demographic, socioeconomic, and health variables. *Health Survey in São Paulo City* (ISA-Capital), São Paulo, Brazil, 2003 and 2015.

Characteristics	2003 [N = 1,667]		2015 [N = 3,184]	
	n	% (95%CI)	n	% (95%CI)
Demographic factors				
Age group (years)				
20-59	795	84.01 (81.91; 85.91)	2,165	81.49 (79.37; 83.44)
60 or more	872	15.99 (14.09; 18.09)	1,019	18.51 (16.56; 20.63)
Gender				
Male	803	45.09 (42.01; 48.20)	1,340	46.26 (44.54; 47.98)
Female	864	54.91 (51.80; 57.99)	1,844	53.74 (52.02; 55.46)
Socioeconomic factors				
Race/Color				
White	1,077	67.51 (63.67; 71.13)	1,629	52.41 (48.71; 56.09)
Black/Mixed-race	506	30.90 (27.33; 34.72)	1,338	45.61 (41.84; 49.43)
Yellow	33	1.59 (0.96; 2.63)	63	1.97 (1.35; 2.88)
Schooling (in full years)				
0-7	959	39.84 (36.83; 42.93)	1,061	25.12 (22.94; 27.44)
8 or more	681	60.16 (57.07; 63.17)	2,113	74.88 (72.56; 76.06)
Health condition				
NCD				
Yes	1,153	59.89 (55.28; 64.34)	2,171	64.58 (62.16; 66.93)
No	514	40.11 (35.66; 44.72)	991	35.42 (33.07; 37.84)
Self-perceived health				
Excellent/Very good	451	36.78 (32.53; 41.24)	581	27.11 (24.48; 29.92)
Good	965	54.49 (50.32; 58.59)	1,600	68.12 (65.39; 70.73)
Bad/Very bad	201	8.74 (6.80; 11.16)	141	4.77 (3.96; 5.73)

95%CI: 95% confidence interval; NCD: noncommunicable chronic disease.

Regarding health conditions, we observed an increasing trend in those affected by NCDs, from 59.89% in 2003 to 64.58% in 2015. We should highlight that health self-perception among individuals also changed during the studied period. The proportion of individuals who rated their health as excellent or very good (36.78% in 2003 and 27.11% in 2015) and bad or very bad (8.74% in 2003 and 4.77% in 2015) decreased, whereas we found an increase in those who described it as good (54.49% in 2003 and 68.12% in 2015) (Table 1).

**Figure 1:**
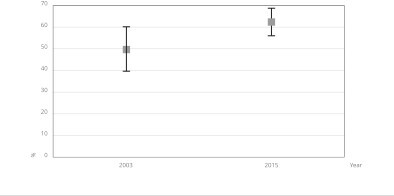
Prevalence (%) of the use of public health service by people who reported health problems in the two weeks before the survey. *Health Survey in São Paulo City* (ISA-Capital), São Paulo, Brazil, 2003 and 2015.

Table 2 and Figure 1 provide information on the frequency of access and use of health care services due to health disorders in the two weeks preceding the survey. We found that the demand for health care in the two weeks preceding the survey increased from 53.77% in 2003 to 64.79% in 2015. Health care use on-demand also increased between 2003 and 2015, from 73.65% to 94.86%. When analyzing health care use by type of service, we found an increasing trend in the use of public services (from 91.25% in 2003 to 95.90% in 2015) and in the use of private services (from 61.85% in 2013 to 93.17% in 2015) as well.

**Table 2:** Access and use of health care services by people who reported health problems in the two weeks before the survey.

Health care use	2003 [N = 1,667]		2015 [N = 3,184]	
	n	% (95%CI)	n	% (95%CI)
Health problem *	448	27.91 (24.05; 32.13)	642	18.95 ** (17.39; 20.62)
Demand for health care ***	249	53.77 (46.97; 60.53)	409	64.79 (60.24; 69.10)
Access and use of health care ^#^	190	73.65 (63.64; 81.70)	389	94.86 ** (91.26; 97.02)
Public	105	91.25 (76.97; 97.02)	128	95.90 (92.38; 97.83)
Private	85	61.85 (48.40; 73.70)	261	93.17 ** (84.35; 97.19)
Satisfaction ^##^	152	77.62 (67.54; 85.26)	285	71.91 (66.17; 77.01)
Public	79	71.91 (56.55; 83.43)	177	64.37 (57.48; 70.71)
Private	73	83.28 (68.18; 92.04)	108	84.45 (75.44; 90.57)
Resolution ^###^	64	32.36 (24.29; 41.64)	205	53.75 ** (46.83; 60.52)
Public	39	36.05 (23.41; 50.97)	138	50.90 (43.81; 57.95)
Private	25	28.71 (18.87; 41.08)	67	58.54 ** (46.83; 69.35)

95%CI: 95% confidence interval.

* Health problem in two weeks prior to the survey;

** Indicates that the differences between the prevalence results are significantly different;

*** Demand for health services to solve health problems;

^#^ Access to and use of health care services at the sought service;

^##^ Satisfaction with the care received at the health service;

^###^ Resolution of the health need among those who resorted to health care.

Satisfaction with received health care tended to decrease, from 77.62% in 2003 to 71.91% in 2015. When we analyzed satisfaction, we found (regarding type of service) a decreasing tendency in public services (from 71.91% in 2003 to 64.37% in 2015) and a stable tendency in private services (83.28% in 2003 to 84.45% in 2015). Regarding the resolution of health problems, we observed an increase in the studied period (32.36% in 2003 and 53.75% in 2015), and this resolution tended to increase in public (36.05% in 2003 and 50.9% in 2015) and private services (28.71% in 2003 and 58.54% in 2015) (Table 2). Regarding the use of public health care, we found an increasing trend in use of services from SUS, from 49.73% to 62.5% between 2003 and 2015 (Figure 1).

Table 3 shows our analyses of the estimated PR regarding access and use of health care services for health disorders in the two weeks preceding the survey according to levels of education. We managed to review the changes in inequities related to the use of public health care between 2003 and 2015.

**Table 3:** Prevalence ratios (PR) of health care use, by education level. *Health Survey in São Paulo City* (ISA-Capital), São Paulo, Brazil, 2003 and 2015.

Characteristics	2003	2015
Adjusted PR * (IC95%)	Adjusted PR * (IC95%)
Health problem **		
8 years and more	1.00	1.00
0-7 years	1.24 (0.99; 1.55)	1.07 (0.90; 1.28)
Demand for health care ***		
8 years and more	1.00	1.00
0-7 years	0.99 (0.75; 1.31)	0.87 (0.74; 1.03)
Access and use of health care ^#^		
8 years and more	1.00	1.00
0-7 years	1.10 (0.84; 1.44)	1.01 (0.95; 1.08)
Satisfaction ^##^		
8 years and more	1.00	1.00
0-7 years	1.07 (0.86; 1.34)	0.95 (0.81; 1.11)
Resolution ^###^		
8 years and more	1.00	1.00
0-7 years	1.09 (0.64; 1.86)	0.91 (0.71; 1.16)
Use of public health service ^§^		
8 years and more	1.00	1.00
0-7 years	2.48 ^§§^ (1.61; 3.81)	1.44 ^§§^ (1.23; 1.68)

95%CI: 95% confidence interval.

* Adjusted for gender and age;

** Health problem in two weeks prior to the survey (C101a. Did you have any health problems in the last 2 weeks?);

*** Demand for health services to solve this health problem (C101i. Did you seek help or talk to someone to solve this health problem?);

^#^ Access to and use of health care services at the service you sought (G111. Did you receive care at the service you sought?);

^##^ Satisfaction with the care received at the health service (G134. What did you think of the care you received?);

^###^ Resolution of the health need among those who resorted to health care (G137. Has your need been resolved?);

^§^ Use of the public health service (G110. Is this health service public or private/private?);

^§§^ Indicates that the results are statistically significant.

In 2003, the use of public health care services was 148% (PR = 2.48; 95% confidence interval - 95%CI: 1.61-3.81) higher among individuals with lower levels of education, whereas, in 2015, analysis found that the use of public health care services was 44% (PR = 1.44; 95%CI: 1.23-1.68) higher among individuals with a lower level of education than among those with higher levels of education. Analysis showed that inequities in the use to public health care services suffered the influence of levels of education in which we observed a strong positive association between these two variables (p < 0.001). Despite this association, the magnitude of disparities in the use to public health care services between individuals with different levels of education decreased in the studied period (Table 3).

## Discussion

The demographic and socioeconomic characteristics observed in this study correspond to changes found in surveys of the Brazilian population ^14^ ^,^ ^15^ ^,^ ^16^ ^,^ ^24^. A higher proportion of older adults and the increase in the number of people who identified themselves as black and mixed-race confirm the results observed in Brazil since the 1980s due to its aging population and the changes in its socially constructed ethnic-racial identification ^25^. The increase in the level of education follows the national trend confirmed by the *Continuous Brazilian National Household Sample Survey* (Continuous PNAD), which found a significant increase in the level of education of the population from 2012 to 2018 ^22^.

Regarding health conditions, the increase in the prevalence of adults with chronic diseases has also been confirmed by the *Global Burden of Diseases* ^26^ and PNS ^14^ and requires attention due to its impact on individuals’ lives and the health care network given the greater need for access and use of health care services ^27^. The decline in the prevalence of excellent or good self-assessments confirms the results found in the PNAD analysis from 1998 to 2013. We found a decrease in positive health self-assessment, especially among populations with lower levels of education. Such a decrease could relate to a better knowledge of each individual’s health status as a result of greater use and access to health care ^15^.

Health system performance characteristics - access, cost, effectiveness, and satisfaction - are increasingly interrelated ^28^. Thus, the analysis of the elements of the use of health systems and services becomes an important object of study.

Analyses of access and use of health care services in 2003 and 2015 found an increase in the demand for services due to health disorders in the two weeks preceding the survey. These results agree with the findings of the PNS ^14^, which also observed an increase in demand for health care in the country, higher among populations with higher levels of education. We should emphasize that health care use partially depend on the morbidity, severity, and urgency of health disorder ^5^. We should consider that the increase in the prevalence of chronic diseases we observed could also explain the increased demand for health care, considering how the progression of acute health disorders affects demand.

Another point worth highlighting is the increase in access and use of health care services among individuals who reported having a demand in the two weeks preceding the survey. A study that used PNAD data from 1998 to 2013 and PNS data confirmed the trend of increased use of health services over the years by all individuals. However, we should stress that despite the increase in access and use of health care services, we also find an increase in inequalities regarding dental services ^29^.

Regarding satisfaction with the care received for health problems in the two weeks preceding the survey (a proxy of health care service quality), this study found that satisfaction remained stably high, with a downward trend regarding public services and stability regarding private services. A study conducted by the PNS in 2013 in the five regions of Brazil found that its Southeast, which has the highest Human Development Index (HDI), had a better evaluation of the care received in hospital services than the other regions of the country ^16^.

We should stress that determinants of potential access are unable to explain effective access because its effects on health and satisfaction (effective access) go beyond the determinants of service use. As mentioned, service use depends on predisposing factors, health needs, and contextual factors, and effective and efficient use depends on individual factors and factors within the health care system that affect the quality of the provided services ^5^.

Regarding the resolution of the health problem by the care received in the two weeks preceding the survey, we observed an increase in private services from 2003 to 2015. Health problem resolution has been studied in other works and consists of health problem resolution and patients and healthcare providers’ satisfaction ^30^. Our results differ from a study conducted in six basic health units (UBS) in a large municipality in the State of São Paulo, which found that users complain about delays in care services and, consequently, in the resolution of their health problems ^31^.

We found a rise in the prevalence of the use of public services from 2003 to 2015. The rise in the use of public services could relate to the significant expansion in the offer of private health insurance with limited coverage during this period, as shown by Bahia et al. ^32^, who found that health insurance companies included public facilities in their network of authorized partners.

The population who used SUS the most was the population with the lowest socioeconomic status in all the studied years. SUS use failed to differ between adults and older adults, confirming a universal system that follows the principle of longitudinal care, in which patients receive care from primary health care (PHC) professionals over time, which is considered a central feature of this level of care and essential to the effectiveness of SUS ^33^.

We observed no inequalities according to education in the prevalence of use of services due to health problems in two weeks prior to the survey, demand for health services to solve this health problem, satisfaction with the care received at the health service, and resolution of health needs among those who resorted to health care. This result may be linked to the structural, operational, and relational dimensions that influence access to health services ^34^. The Municipality of São Paulo, as a structural dimension, shows a unique socioeconomic context that impacts the living conditions of its resident population; as an operational dimension, it has a complex network of organized services that permeate all levels of health care; and as a relational dimension, it shows a diversity of perceptions, beliefs, and values, which may imply the understanding of health-disease and the search for services.

Another important point we should highlight based on the absence of inequalities for these analyzed factors is the success of the SUS as a social inclusion policy. Results show that health services in São Paulo are more equitable and guarantee access despite its population size and social diversity.

The analysis of the prevalence of access to and use of health care services by level of education, only showed a statistically significant association with the use of public services. We found that individuals with less than eight years of schooling had greater access and use of public services than those with more than eight years of schooling. This result resembles other studies, which found a greater use of public services in less educated individuals ^23^ ^,^ ^35^, a situation that may relate to their lower ability to pay for health services privates or by achieving equity in health.

Comparing 2003 and 2015, we found a reduction in inequalities in the use of public health services according to levels of education. The role of SUS in reducing inequities and its equity factor stands out. Despite its problems, especially in the depletion of the system over the years, SUS has ensured health care for all the Brazilian population, especially in São Paulo, during the three decades of its history, as per our results. However, it faces challenges to solve the population’s health problems.

The complexity and breadth of the system pose challenges to health services in São Paulo. A system that still faces unresolved basic and primary problems, such as a lack of basic sanitation. As in most Brazilian cities, the inhabitants of the most diverse and distant outlying districts are forced to commute daily in a low-quality transportation system. We are unable to deny the contribution of territory and segregation processes to the production and reproduction of vulnerabilities and inequalities affecting most urban areas, especially in the periphery of metropolitan areas such as São Paulo, in which intra-urban inequalities and the effects of segregation have tended to worsen as a result of social transformations, the pandemic crisis, and the reorientation of urban policies ^36^.

According to Esposti et al. ^37^ since access to health care alone is unable to contribute to the reduction of health inequalities, it is necessary to set intersectoral partnerships to promote better living conditions for populations.

This study shows the role of SUS in the last 15 years in São Paulo, one of the most socially unequal cities in the world ^38^, with a complex health care network.

The inequalities in São Paulo became even more evident in the COVID-19 pandemic, highlighting the importance of SUS in playing a leading role in caring for the victims of the disease ^3^ and in vaccinating the population. SUS has provided the best responses to this crisis, ranging from direct assistance to health care users to the combined operation of public laboratories and the epidemiological surveillance by the Brazilian Ministry of Health ^39^. The points discussed above should guide a public agenda to restructure the São Paulo municipal government after the pandemic.

We should point out that the answers to the ISA-Capital survey are self-reported and may contain errors in the classification of participants’ answers. Moreover, we conducted our analyses up to 2015, the most recent period with available data.

However, we should emphasize that these data come from a population-based survey, contributing to information that represents the extent of the problem in the most populous Brazilian megalopolis and one of the most socially unequal municipalities in the world. Moreover, ISA-Capital can measure information on all health care services used by the population, including different types of public and private services, thus serving to monitor inequalities.

## Conclusions

We observed no social inequities in health care use among those who reported health disorders in the two weeks preceding the survey in the Municipality of São Paulo for issues related to demand, access, satisfaction, and resolution according to levels of education. Results show important progress in the use and resolution of health problems, as well as in the increase of the prevalence of public services use in 2003 and 2015. The population who most frequently used SUS was that with the lowest socioeconomic status in all studied years. The role of SUS is to reduce inequalities, and we found that the equity factor stands out. The higher prevalence of public health care use individuals with lower levels of education evince the work of the SUS in the Municipality of São Paulo. We should also consider that despite the increase in the resolution of health problems, we found a trend toward a reduction in satisfaction with the care received in public health services (although not statistically significant), indicating the importance of further studies to further develop and monitor the analysis of the quality of provided services.
